# Toward a generalized Bienenstock-Cooper-Munro rule for spatiotemporal learning via triplet-STDP in memristive devices

**DOI:** 10.1038/s41467-020-15158-3

**Published:** 2020-03-20

**Authors:** Zhongqiang Wang, Tao Zeng, Yanyun Ren, Ya Lin, Haiyang Xu, Xiaoning Zhao, Yichun Liu, Daniele Ielmini

**Affiliations:** 1 Key Laboratory for UV Light-Emitting Materials and Technology (Northeast Normal University), Ministry of Education, Renmin Street, 5268 Changchun, China; 2 Dipartimento di Elettronica, Informazione e Bioingegneria, Politecnico di Milano, Piazza L. da Vinci 32, 20133 Milano, Italy

**Keywords:** Engineering, Electrical and electronic engineering, Electronic devices

## Abstract

The close replication of synaptic functions is an important objective for achieving a highly realistic memristor-based cognitive computation. The emulation of neurobiological learning rules may allow the development of neuromorphic systems that continuously learn without supervision. In this work, the Bienenstock-Cooper-Munro learning rule, as a typical case of spike-rate-dependent plasticity, is mimicked using a generalized triplet-spike-timing-dependent plasticity scheme in a WO_3−x_ memristive synapse. It demonstrates both presynaptic and postsynaptic activities and remedies the absence of the enhanced depression effect in the depression region, allowing a better description of the biological counterpart. The threshold sliding effect of Bienenstock-Cooper-Munro rule is realized using a history-dependent property of the second-order memristor. Rate-based orientation selectivity is demonstrated in a simulated feedforward memristive network with this generalized Bienenstock-Cooper-Munro framework. These findings provide a feasible approach for mimicking Bienenstock-Cooper-Munro learning rules in memristors, and support the applications of spatiotemporal coding and learning using memristive networks.

## Abstract (web summary)

Designing reliable and energy efficient neuromorphic computing systems for spatiotemporal coding remains a challenge. Here, the authors demonstrate a type of spike-rate-dependent plasticity based on a triplet learning scheme in a WO_3−x_-based second-order memristor network for spatiotemporal patterns.

## Introduction

Brain-inspired neuromorphic computing systems are attracting strong interest because of their massive parallelism, high energy efficiency, good error tolerance, and good ability to implement cognitive functions^1–6^. Hardware implementations of neuromorphic computing can take advantage of novel nanodevices that emulate the biological synapses with inherent learning functions^7–13^. The two-terminal memristor is widely recognized as a promising technology with which to mimic the biological synapse because of its functional resemblance to the biological counterpart^14–19^. The biorealistic realization of synaptic plasticity in the memristor is considered to be an important step toward realizing an artificial synapse with high accuracy. There have been many efforts to demonstrate basic synaptic learning functions using single and paired spikes, for example, long-term/short-term plasticity, spike-timing-dependent plasticity (STDP), and paired-pulse facilitation (PPF)/depression^20–27^. In fact, the stimulation mode of a spike train that contains plentiful spikes is a more general case than the single spike or paired spikes, and is produced by a neuron receiving multiple spikes from other connected neurons^28^. The information contained in a spike train allows specific advanced plasticity within a synapse that is referred to as spike-rate-dependent plasticity (SRDP)^29^.

The Bienenstock-Cooper-Munro (BCM) learning rule is an important type of SRDP beyond the Hebbian learning rule and describes history-dependent synaptic modification. In the BCM framework, the high/low spike rate of a train can result in the potentiation/depression of the synaptic weight depending on whether the spike rate is higher than a threshold (*θ*)^30–33^. For the memristor-based artificial synapse, several groups have demonstrated BCM rules using rate-based presynaptic spikes, which have led to advances in the field^34–36^. These results show that the absolute change in the synaptic weight (i.e., the conductance change of the memristor, *|*Δ*G*_*c*_|) has a monotonic dependence on the spike rates in both the depression region (Δ*G*_*c*_ < 0) and potentiation region (Δ*G*_*c*_ > 0). However, such a monotonic change is different from the original BCM rule in neurobiology; that is, there should exist non-monotonic behavior (i.e., an enhanced depression effect (EDE)) in the depression region^30–33,37,38^. Additionally, previous memristor studies lack the following essential features: first, the lack of a multiplicative term between presynaptic and postsynaptic activities, and second the short-term modification^34–36^. This also marks a significant inconsistency with the biological BCM learning.

According to a theoretical model of Pfister et al., it is expected that the use of triplet-STDP, instead of common rate-based presynaptic spikes, allows this issue to be solved^39,40^. Furthermore, the BCM rule can be generalized by the long-term triplet-STDP, thereby allowing higher-order spatiotemporal recognition in the visual cortex, for example, rate-based orientation selectivity^39^. Triplet-STDP means that a third spike, either presynaptic or postsynaptic, is introduced into the standard pair-STDP^33,40–42^. Importantly, in addition to the paired term contribution in pair spikes, a previous spike (presynaptic or postsynaptic) also causes the contribution of a triplet term in the triplet-STDP^33,41,42^. The relationship between the paired term and triplet term contributions provides the multiplicative correlations between presynaptic and postsynaptic activities, which is an essential requisite for BCM learning. There are two types of triplet-STDP in neuroscience: the first-spike-dominating model and last-spike-dominating model proposed by Froemke et al. and Wang et al., respectively^41,42^. Progress has been made in emulating these two types of triplet-STDP using first-order and second-order memristors^36,43–45^. However, the generalization from triplet-STDP to the BCM learning rule has not yet been experimentally demonstrated in memristors. Additionally, high-order spatiotemporal recognition that relies on generalized BCM learning rules has rarely been reported.

The present work presents the demonstration of generalized BCM learning rules using the last-spike-dominating triplet-STDP in a WO_3−x_-based second-order memristor. The second-order memristor has physical behavior similar to Ca^2+^ dynamics in the bioneural network, which allows the emulation of rate-based plasticity naturally^16,34,46^. The EDE, which was typically missing in previous studies, is achieved using a long-term triplet-STDP scheme. Our experimental results are highly consistent with the mathematical model of the BCM framework in a biological system. Additionally, rate-based orientation selectivity is demonstrated on the basis of such a generalized triplet-STDP-based BCM learning rule, showing its strong potential in high-order spatiotemporal recognition.

## Results

### Motivation and WO_3−x_ second-order memristor

**Fig. 1:**
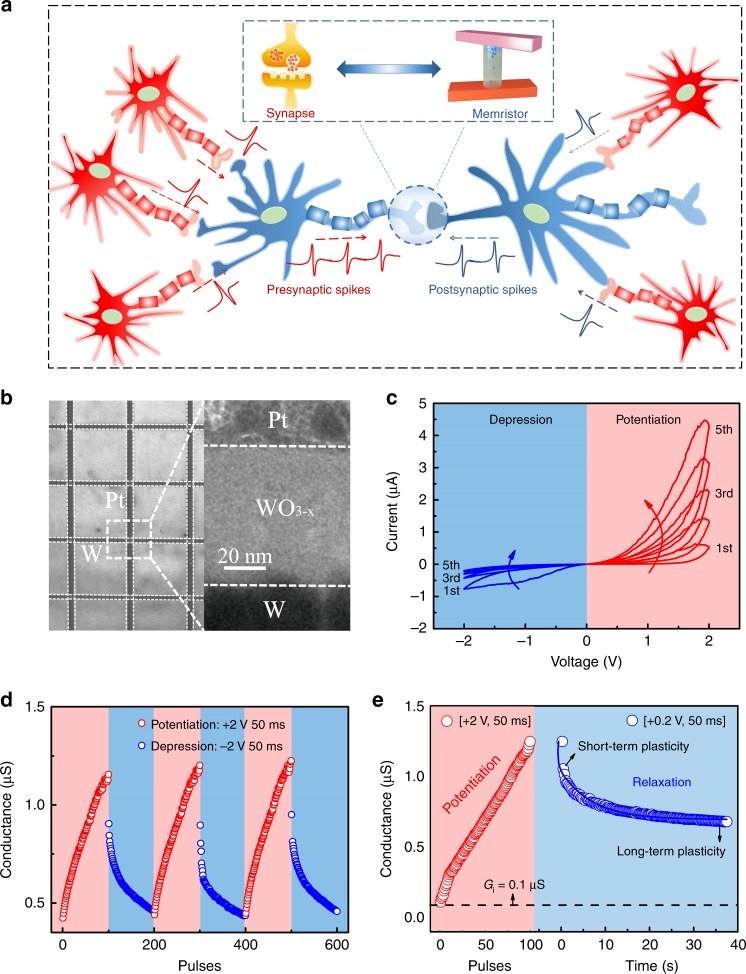
Artificial synapse based on the WO_3−x_ second-order memristor. **a** Schematic of spike trains in a biologic system, produced by a neuron receiving multiple spikes from other connected neurons. **b** Scanning electron microscopy image of the memristor crossbar array and a transmission electron microscopy image of the cross-section of the Pt/WO_3−x_/W memristor. **c** I–V curve of the memristor under a continuous positive sweep (0 to 2 V) and negative sweep (0 to −2 V). **d** Potentiation and depression of conductance G_c_ caused by the repeated stimulation of 100 positive pulses [+2 V, 50 ms] and 100 negative pulses [−2 V, 50 ms]. All the data of *G*_*c*_ were collected 1 s after the stimulation using a reading pulse [+0.2 V, 50 ms], which represents the long-term plasticity. **e** Spontaneous decay of conductance *G*_*c*_ after the potentiation process. This decay process may be related to the diffusion of oxygen ions, which is an indicator of a second-order memristor. Here, the device was operated from an initial conductance *G*_*i*_ of 0.1 µS. The relaxation process was monitored under a read pulse [+0.2 V, 50 ms].

Figure 1a depicts the motivation for a biologically plausible triplet-STDP-based BCM learning rule in memristive hardware. Generally, neurons in a neurophysiological system interact by exchanging spike trains, that is, sequences of pre- and postsynaptic spikes. Because of the diversity of the spike trains, the synaptic modification is not only determined by the timing between paired spike, as in the case of common STDP, but also affected by the details of the spike pattern, such as the spike train rate^23,29,47^. A relatively simple model of pattern-dependent plasticity is triplet-STDP^41,42^. Typical BCM learning rules can then be generalized based on triplet-STDP, which also allows rate-based orientation selectivity for high-order spatiotemporal functions.

To demonstrate the aforementioned synaptic functions, we considered the WO_3−x_-based second-order memristors illustrated in Fig. 1b, which consist of a Pt/WO_3−x_/W sandwich structure prepared in crossbar arrays using a sputtering deposition technique. Figure 1c shows the I–V curve of the WO_3−x_-based memristor, gradually increases and decreases with positive- and negative-bias sweeps on the top Pt electrode. The conductance *G*_*c*_ can be regarded as the synaptic weight. The asymmetric I–V curve of Fig. 1c also indicates the existence of Schottky barrier at Pt/WO_3−x_ interface. This memristive behavior is similar to the nonlinear transmission of a biological synapse, thereby allowing for a continuous adjustment of synaptic weight. Analogous to biological spikes, a series of positive and negative pulses elicits the consecutive potentiation and depression of our memristive synapse, as shown in Fig. 1d. Additionally, the potentiation state can spontaneously decay to a middle state, as shown in Fig. 1e, which is a clear indicator of the second-order memristor (Supplementary Fig. 1 and Note 1)^16,17,34^. Such behavior is equivalent to a transition from short-term plasticity to long-term plasticity by repeated stimulation^21^. The memristor dynamics are caused by oxygen-ion diffusion that resembles biological Ca^2+^ dynamics^15,16,21,24^. Du et al. previously developed a WO_x_ memristive device with a Pd/WO_x_/W structure where the switching mechanism was attributed to the modification of a relative area of the conducting channel^34^. In our work, instead, the Schottky barrier of the Pt/WO_3−x_ interface and its modulation induced by the drift and diffusion of oxygen ions can account for the memristive mechanism (Supplementary Fig. 1 and Note 1). The different device fabrication methods and electrodes may be responsible for the different memristive behaviors. A similar Schottky barrier based memristive mechanism was reported in our previous work and other literature^22,36,44,48^.

### Synaptic adaptation function emulated by rate-based postsynaptic spikes

**Fig. 2:**
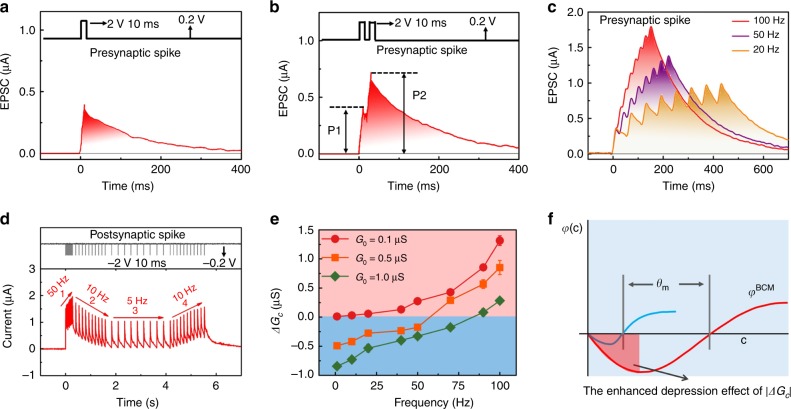
Emulation of the rate-based synaptic adaptation function using postsynaptic spikes in the Pt/WO_3−x_/W memristors. **a, b** Synaptic EPSC and PPF functions triggered by a presynaptic spike [2 V, 10 ms] and paired spikes. **c** Dependence of the EPSC amplitude on the rate of presynaptic spike trains (20, 50, and 100 Hz). Eight pulses [2 V, 10 ms] were used to stimulate the device. **d** Response of the synaptic weight (conductance *G*_*c*_) to a group of postsynaptic spike trains with a frequency sequence (50 Hz → 10 Hz → 5 Hz → 10 Hz). The pulse [−2V, 10 ms] on the bottom electrode as the postsynaptic spike. **e** Sliding threshold effect of the history-dependent synaptic adaptation function with different *G*_0_. Herein, the experienced *G*_0_ was activated to three levels starting from a fixed initial conductance of *G*_*i*_ = 0.1µS. For the case of *G*_0_ = 0.5 µS and 1.0 µS, the stimulation was conducted using a pulse amplitude of −2V and pulse width of 10 ms with postsynaptic spike rates of 20 Hz and 50 Hz, respectively. The peak of temporary conductance potentiation (*G*_peak_) was collected to calculate Δ*G*_*c*_ = *G*_peak_ − *G*_0_, which represents a type of short-term plasticity. **f** Biological BCM curve. The vertical and horizontal axes are the weight modification *φ(c)* and postsynaptic firing rate *c*, respectively. According to the BCM function of *φ*^BCM^, the depression/potentiation of *φ(c)* occurs as the firing rate is lower/higher than modification threshold *θ*_*m*_. In particular, the parameter *θ*_*m*_ is adaptive to the experienced activity: the synapse which experienced a period of inactivity would follow the blue curve with smaller *θ*_*m*_, whereas the synapse that experienced a period of enhanced activity would follow the red curve. Reproduced with permission^32^. Copyright 2012, Nature Pub. Group. The red shaded area indicates an EDE of |Δ*G*_*c*_| in the depression region, which was usually absent in previous memristor-based BCM studies.

The synaptic adaptation function was mimicked using a second-order memristive effect in the WO_3−x_ device. Figure 2a shows the current response of the memristive device under a single presynaptic spike [2 V, 10 ms]. The figure shows that the presynaptic spike triggers an abrupt increase in current followed by a decay to the initial state within 400 ms, which is similar to the behavior of the excitatory postsynaptic current (EPSC) of the biological synapse^21,24^. The similarity with the biological EPSC behavior is caused by oxygen-ion drift and diffusion in our memristor paralleling the influx and extrusion of Ca^2+^ through the synaptic cell^24,49^. It is noted in Fig. 2a that there is not an abrupt decrease in current observed, when the stimulation pulse of 2 V is changed to the monitoring pulse of 0.2 V. One of the possible reasons is the electric double-layer (EDL) capacitance, which is discussed in a recent paper by Yang et al.^50^. In a biological system, the Ca^2+^ dynamics allow correlation between paired spikes, where the residual Ca^2+^ induced by the first spike enhances the overall Ca^2+^ concentration generated by the second spike, thus resulting in PPF. The PPF function is critical for a synapse to make correlations between the temporal spike pair. Based on the similarity between oxygen-ion dynamics and Ca^2+^ dynamics, we can demonstrate PPF, as shown in Fig. 2b. When the second stimulation comes before the first EPSC disappears completely, their overlap can effectively suppress the diffusion of oxygen ions. This promotes the more effective accumulation of oxygen ions on the Pt/WO_3−x_ interface, which leads to a larger conductance change. Thus, the peak value of EPSC induced by the second spike P2 is clearly higher than that of the first-spike P1, and a longer interval between presynaptic spikes gradually decreases the facilitation of the second spike (Supplementary Fig. 2). In fact, the EPSC evoked by a single spike results in a temporary effect (i.e., short-term plasticity), whereas the PPF with sufficiently large spikes results in a permanent effect (i.e., conversion from short-term plasticity to long-term plasticity). The PPF effect can also be extended to the SRDP using spike trains instead of spike pairs, as shown in Fig. 2c. In this case, the increase in EPSC amplitude depends on not only the presynaptic spike number but also the spike rate. Spikes with a higher rate (i.e., a shorter interval) result in a much larger EPSC amplitude, similar to the case of SRDP in the biological synapse^51–53^.

History-dependent plasticity is an important behavior for the synaptic adaptation function. To verify the feasibility of history-dependent plasticity for our memristor, we applied a pulse [−2V, 10 ms] to the bottom electrode as the postsynaptic spike in Fig. 2d, including four distinct phases. In the first phase, a postsynaptic spike train at a relatively high frequency (50 Hz) increased the conductance in the memristor. In the second phase, the lower frequency (10 Hz) of the spike train decreased the conductance (i.e., synaptic depression). A spike train of 5 Hz in the third phase and a final spike train of 10 Hz applied in the fourth phase increased the conductance again (i.e., synaptic potentiation). It is interesting to note that the second and fourth phases induced opposing conductance changes even though the same spike trains of 10 Hz were used. In this experiment, the conductance state after stimulation at 50 and 5 Hz can be regarded as the experienced conductance (*G*_0_); that is, different ‘experiences’ or ‘histories.’ The postsynaptic spike train of 10 Hz can thus induce depression or potentiation depending on the experienced *G*_0_ activated by higher (50 Hz) or lower (5 Hz) frequencies, thereby indicating the realization of history-dependent plasticity.

The postsynaptic spike-rate-based synaptic adaptation function was further implemented in WO_3−x_ memristors by changing the learning experience, hence *G*_0_, as illustrated in Fig. 2e. Spiking at relatively low and high frequencies generally results in depression and potentiation, respectively, in line with the results in Fig. 2d. Additionally, the value of *G*_0_ dictates the threshold rate *θ*, which shows a formalistic similarity with the sliding threshold effect of the BCM rule in^30–33^. For this reason, previous studies demonstrated the BCM learning by the implementation of results similar to Fig. 2e^34–36^. Strictly speaking, however, such synaptic adaptation function is not fully equivalent to the biological BCM rule, given three fundamental differences between them: (i) The observed synaptic adaptation genrally consists of a temporary short-term modification of the synaptic weight, while BCM learning should refer to long-term potentiation/depression (LTP/LTD) according to the literature^30–33^. (ii) In BCM learning, the synaptic weight is a function of both the presynaptic and postsynaptic neuron activities and also requires a multiplicative relationship between them. On the other hand, only postsynaptic spikes were used in the synaptic adaptation function implemented in our work and previous studies^34–36^. (iii) Note that conductance change Δ*G*_*c*_ is a monotonic function of the spike rate in both the depression and potentiation regions, which is obviously different from the biological BCM model of Fig. 2f^32^. In the depression region, the depression effect should first enhance (i.e., |Δ*G*_*c*_| increases) at a relatively low spike rate and then weaken as the spike rate increases; that is, the EDE in the depression region (the red shaded area in Fig. 2f) was missing in the existing implementation of the BCM rule using rate-based postsynaptic spikes (Fig. 2e)^34–36^. The absence of the EDE can be understood in that there are only two competitive factors that determine synaptic change Δ*G*_*c*_: the spontaneous forgetting effect of the experienced *G*_0_ and the potentiation effect induced by the presynaptic spike train^34–36^. There is no other rate-based depression effect that can assist the forgetting effect to induce the EDE. It is essential to remedy this EDE region in memristors to closely approximate the biological synapse; however, there is a lack of related studies.

### Triplet-STDP and generalized BCM learning rule

**Fig. 3:**
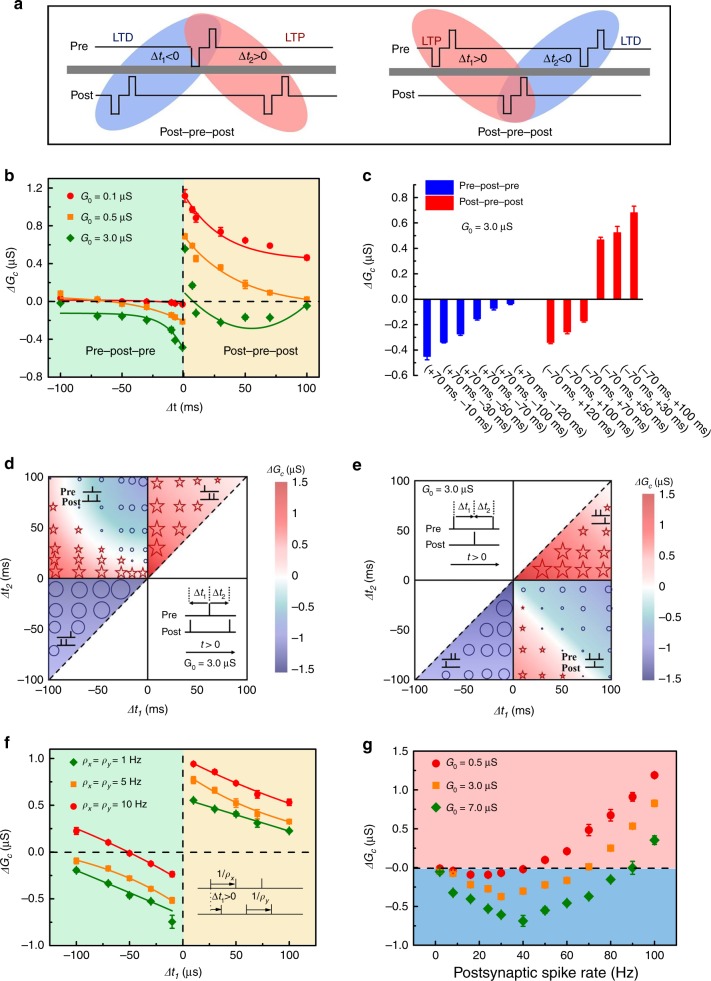
Demonstration of triplet-STDP and its related BCM learning rules in Pt/WO_3−x_/W memristors. **a** Schematic of the typical ‘post-pre-post’ and ‘pre-post-pre’ triplets, which can be simplified as a superposition of the LTP and LTD processes. Each pre- or postsynaptic spike comprises two pulses with amplitude *V*^+^/*V*^*−*^ = 2 V/−2 V and duration 50 ms. Taking the former as an example, the LTD process activated in the first ‘post-pre’ pair with spike timing of Δ*t*_1_ < 0 is followed by the LTP process induced by the second ‘pre-post’ pair with spike timing of Δ*t*_2_ > 0. **b** Synaptic modification of triplet-STDP in the ‘post-pre-post’ and ‘pre-post-pre’ sequences using symmetrical spike timing |Δ*t*_1_| = |Δ*t*_2_|, with three levels of the initial *G*_0_ considered as the learning experiences (i.e., 0.1, 0.5, and 3.0 µS). **c** Triplet-STDP with asymmetrical spike timing. Red column: Δ*t*_1_ = −70 ms, Δ*t*_2_ is from 10 to 120 ms in the ‘post-pre-post’ sequence; Blue column: Δ*t*_1_ = +70 ms, Δ*t*_2_ is from −10 to −120 ms in the ‘pre-post-pre’ sequence. **d, e** Summaries of triplet-STDP results in our experiments, where potentiation or depression with different synaptic weights is obtained using different spike sequences and different timing intervals. The insets show the schematic of ‘post-pre-post’ and ‘pre-post-pre’ sequences. Here, both the size of symbols and the background color indicate the magnitude of Δ*G*_*c*_. A relatively high *G*_*0*_ of 3.0 µS was adopted for the measurements of Fig. 3 (**d, e**) to highlight the history-dependent characteristics. **f** The dependence of Δ*G*_*c*_ on both the presynaptic spike rate *ρ*_*x*_ and postsynaptic spike rate *ρ*_*y*_. The schematic of the operation signal is shown in the inset, in which three pairs of presynaptic spike and postsynaptic spikes were used. **g** Triplet-STDP-based BCM learning rules with the EDE in the low-frequency region and the threshold sliding effect, which is highly similar to the biological BCM curve.

Different from previous implementations using common rate-based postsynaptic spikes, in theory, the BCM learning rule can be closely replicated using a generalized model based on triplet-STDP^39,40^. This generalized triplet-STDP-based BCM rule is experimentally demonstrated in our memristive devices. The long-term paired-STDP was first implemented on an experimental basis and as a comparison for triplet-STDP. For the paired-STDP, the time delay between paired spikes (i.e., Δ*t* = *t*_post_ — *t*_pre_) determines whether the LTP at Δ*t* > 0 (i.e., the presynaptic spike is earlier than the postsynaptic spike) or LTD at Δ*t* < 0 occurs, which was also demonstrated in our WO_3−x_-based memristors (Supplementary Fig. 3 and Note 2). For the long-term triplet-STDP, the spike train can be assumed to be the combination of two spike pairing events, and the synaptic modification can thus be regarded as the integration of LTP and LTD processes induced by these two events^42^. Additionally, the triplet term induced by the previous spike to the paired spikes also needs to be taken into account. Figure 3a illustrates two typical triplets with ‘post-pre-post’ and ‘pre-post-pre’ sequences. For the ‘post-pre-post’ triplet, the LTD process is induced by the first-spike pairing (‘post-pre’, Δ*t*_1_ < 0), which is followed by an LTP process induced by the second spike pairing (‘pre-post’, Δ*t*_2_ > 0). For the ‘pre-post-pre’ triplet, the LTP process is activated before the LTD process. Hereafter, the pair (Δ*t*_1_, Δ*t*_2_) is used to denote the spike timing in triplets. This means that the ‘post-pre-post’ triplet always has the timing of Δ*t*_1_ < 0 and Δ*t*_2_ > 0, whereas the ‘pre-post-pre’ triplet has the timing of Δ*t*_1_ > 0 and Δ*t*_2_ < 0. In the triplet-STDP scheme, each spike applied on the memristor consists of a pair of pulses [*V*^+^/*V*^*−*^ = 2 V/−2 V, 50 ms] as illustrated in Fig. 3a and in our previous work^21,22^. Refer to Supplementary Fig. 3 for details of the spike design; a delay time of 60 s was introduced to ensure the readout of long-term conductance change before and after the spikes^21^.

The synaptic modification of triplet-STDP was examined with the symmetrical spike timing of the LTP and LTD processes (i.e., |Δ*t*_1_| = |Δ*t*_2_|), as shown in Fig. 3b. For the ‘post-pre-post’ triplet, the obvious potentiation of Δ*G*_*c*_ was observed in the case of *G*_0_ = 0.1 µS (see the red circle in Fig. 3b), whereas the potentiation exponentially decreased with increasing spike timing Δ*t*. For the ‘pre-post-pre’ triplet, there was no notable change in Δ*G*_*c*_ after the stimulation in the case of *G*_0_ = 0.1 µS, which indicates that the LTP process was canceled by the following LTD process. In fact, the asymmetrical result of these two triplet sequences cannot be understood by simply considering the competition of LTP and LTD in the paired-STDP model^39,40^. In that interpretation, the LTP and LTD processes should counteract each other in both triplets regardless of their sequence. Interestingly, this result presented a demonstration of the last-spike-dominating triplet-STDP, and a similar finding was reported in a neurobiological study published by Wang et al.^42^. Furthermore, the effect of the long-term learning experience (i.e., different *G*_0_ measured in long-term plasticity) on triplet-STDP can be observed in Fig. 3b. Our previous work indicates that the stimulation from an intermediate state with long-term learning experience can induce a larger Δ*G*_*c*_^21^. The physical mechanism may be related to the metastable local structure (e.g., unstable interstitial oxygen ions) with a lower energy barrier for further defect migration^21^. For the case *G*_0_ = 0.5 µS, the potentiation was reduced overall, even to the extent that the depression appeared for a relatively long spike timing in the ‘post-pre-post’ triplet, whereas the depression strengthened for higher G_0_ in the ‘pre-post-pre’ triplet. In particular, when *G*_0_ increased to 3.0 µS, Δ*G*_*c*_ in the ‘post-pre-post’ triplet was no longer a monotonic function of the spike interval (Δ*t*_1_ = Δ*t*_2_). The potentiation of Δ*G*_*c*_ happened for a relatively short interval time (Δ*t* < 8 ms), while a longer interval time induced the depression of Δ*G*_*c*_, as illustrated by the green-diamond data in Fig. 3b. This behavior is similar to the biological BCM learning rule when converting the interval time into the frequency^39,40^. This provides an experimental foundation with which to remedy the absence of the EDE region in the BCM learning rule, which will be demonstrated later. As discussed previously, there are only two competitive factors in determining *ΔG*_*c*_ in the BCM rule implemented using common rate-based postsynaptic spikes, which leads to the absence of the EDE region. By contrast, an additional depression effect of the LTD process is introduced in triplet-STDP. Together with the forgetting effect of experienced *G*_0_ and the potentiation effect of the LTP process, there is a third competitive factor in triplet-STDP. With the help of the forgetting effect of high *G*_0_, this third factor, that is, the EDE of the LTD process (Supplementary Fig. 4), could make up for the absence of the EDE region, as illustrated in Fig. 2f. Meanwhile, the asymmetrical spike-timing intervals played a critical role in the competition of LTP and LTD processes, as shown in Fig. 3c. In the ‘post-pre-post’ case, Δ*G*_*c*_ transformed from potentiation to depression as the interval of the LTP process (*Δt*_2_) increased from 10 to 120 ms whereas the interval of the LTD process (Δ*t*_1_ = −70 ms) was kept constant. Similarly, in the ‘pre-post-pre’ case, the depression of Δ*G*_*c*_ weakened with increasing |Δ*t*_2_| and while keeping Δ*t*_1_ = 70 ms constant.

Figure 3d, e summarize the data of the last-spike-dominating triplet-STDP with varying Δt_1_ and Δt_2_ using a colored background to show Δ*G*_*c*_. In addition to the mentioned ‘post-pre-post’ and ‘pre-post-pre’ cases, four other patterns (i.e., two pre-spikes (post-spikes) occur before and after a single post-spike (pre-spike)) were included, as shown in quadrants I and III of Fig. 3d, e. Generally, the potentiation effect was observed in quadrant I because the pre-spike was always applied before the post-spike regardless of the spike number, similar to the LTP process of paired-STDP. By contrast, the depression effect was dominant in quadrant III, similar to the LTD process of paired-STDP. For quadrants II and IV, the synaptic weight *G*_*c*_ can be switched from depression to potentiation by adjusting the timing of Δ*t*_1_ and Δ*t*_2_, which is in accordance with the results of Fig. 3b, c. The results shown in Fig. 3d, e also indicate the possible dependence of Δ*G*_*c*_ on the interval time between two postsynaptic spikes Δ*t*_*o*_ = *t’*_post_ − *t*_post_ and that between two presynaptic spikes Δ*t*_*r*_ = *t’*_pre_ − *t*_pre_, where *t*_post_, *t’*_post_, *t*_pre_, and *t’*_pre_ are the times of the two postsynaptic spikes and two presynaptic spikes. In fact, the dependence of Δ*G*_*c*_ on both the presynaptic spike rate *ρ*_*x*_ = 1/Δ*t*_*r*_ and postsynaptic spike rate *ρ*_*y*_ = 1/Δ*t*_*o*_ can be expected, which is also an essential feature in triplet-STDP^29,39–42^. The dependence of LTP/LTD on both the presynaptic and postsynaptic spike frequency was experimentally measured, as shown in Fig. 3f. The *ρ*_*x*_ and *ρ*_*y*_ share the same value for the spike frequency in this measurement. Data indicate that the LTP increases as the spike frequency increasing from 1 Hz to 10 Hz, while the LTD decreases at increasing the spike frequency. Such dependence on both the presynaptic and postsynaptic spike frequency is consistent with the biological triplet-STDP while it fails to appear in paired-STDP^29,39–42^.

Figure 3d, e can guide us in designing a rational triplet-STDP scheme (e.g., choosing a proper relationship between Δ*t*_1_ and Δ*t*_2_) to fully implement the BCM learning rule. A typical example is shown in Fig. 3g, where the diagonal line of quadrant II (i.e., the ‘post-pre-post’ triplet with |Δ*t*_1_| = |Δ*t*_2_|) is chosen, the devices with high G_0_ (i.e., 0.5, 3.0, and 7.0 µS) were used, and the spike timing sum of |Δ*t*_1_| and |Δ*t*_2_| was treated as the frequency of postsynaptic spikes using *ρ*_*y*_ = 1/(|Δ*t*_1_| + |Δ*t*_2_|). Figure 3f clearly shows the transition of Δ*G*_*c*_ from depression to potentiation with the spike rate increasing to a threshold value. In particular, the EDE region in the BCM, which was typically absent in previous implementations using the common rate-based postsynaptic spikes^34–36^, was demonstrated for our memristive synapse. Furthermore, the threshold sliding effect with different learning experiences is shown in Fig. 3g, in which the increase in *G*_0_ from 0.5 to 7.0 µS resulted in a rise of the threshold frequency from 40 to 90 Hz. The forgetting effect thus strengthened with learning experience *G*_0_ increasing to larger values. Our results are therefore in good agreement with the BCM curve of Fig. 2f, thereby indicating the close reproduction of the BCM learning rule. In fact, if using asymmetrical spike timing with a fixed difference (e.g., |Δ*t*_1_| − |Δ*t*_2_| = 20 ms) in quadrant II, the triplet-STDP-based BCM rule can also be demonstrated (Supplementary Fig. 5). It is believed that the aforementioned triplet-STDP methods can also be used to obtain the generalized BCM learning rule in other second-order memristors. It is necessary to mention that there are still certain differences between the memristive implementation and the real biological BCM rule. For instance, in the biological BCM rule there is no synaptic weight change for *ρ*_*y*_ = 0 and for any value of *ρ*_*x*_, which is instead not the case in our memristive implementation (Fig. 3g). This is mainly because the equivalent pre-/postsynaptic pulses were designed to be equal to simplify the circuit operation, which may be different from the real biological synapse. Anyway, the realization of generalized triplet-STDP-based BCM rule with a long-term nature in memristors has potential applications in high-order spatiotemporal pattern recognition.

### Simulation of spatiotemporal selectivity

**Fig. 4:**
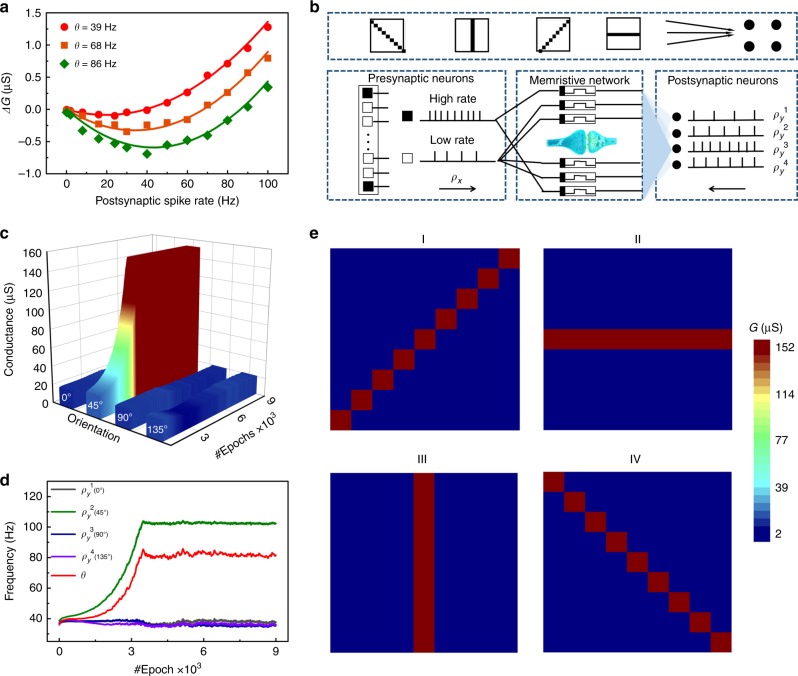
Simulation of rate-based orientation selectivity using the triplet-STDP-based BCM model of Pt/WO_3−x_/W memristors. **a** Experimental data (dots) and calculated curves (lines) of BCM theory, which both have a good fit. **b** Schematic illustration of the memristor-based feedforward neural network. Presynaptic spikes are generally Poisson spike trains with a mean rate <*ρ*_*x*_>. **c** The *G*^1^ evolution of the extracted synapses only for the four orientations with the learning epochs. *G*^1^ denotes the synaptic weight of the synapses connected to the 1st postsynaptic neuron. **d** Evolution of *ρ*_*y*_^1^ corresponding to the four orientation bars with the learning epochs. **e** Color maps of the final synaptic weights in the 9 × 9 memristor arrays which reveal the orientation selectivity for four postsynaptic neurons.

The realization of high-order spatiotemporal functions in memristive devices is a critical step in building a neuromorphic system that mimics the biological brain. Sun et al. and Wang et al. demonstrated the localization of sound by establishing correlations between the sound azimuth and spike timing^54,55^. Spatiotemporal selectivity (e.g., rate-based orientation selectivity) is another type of classical spatiotemporal function in the visual cortex; however, it has not yet been addressed in memristors. According to the studies of Pfister et al.^39,40^, rate-based orientation selectivity can be expected, following the results of long-term triplet-STDP-based BCM learning rules. A mathematical simulation of the experimental data of the BCM rule is necessary to build the neural network for spatiotemporal selectivity. The characteristics of *ρ*_*x*_- and *ρ*_*y*_-dependence (Fig. 3f, g) provided the experimental basis for relating triplet-STDP to the BCM learning rule. The mathematical model of BCM learning can be expressed by refs. ^23,39,40,56,57^:

1 $$dG_c/dt = \rho _x\varphi \left( {\rho _y,\theta } \right),$$

where *ρ*_*x*_, *ρ*_*y*_ are the rates of presynaptic spikes and postsynaptic spikes, respectively and *θ* is the threshold rate. Depression of the synaptic weight (Δ*G*_*c*_ < 0) occurs if *φ*(*ρ*_*y*_ < *θ*, *θ*) < 0, whereas potentiation of the synaptic weight (Δ*G*_*c*_ > 0) occurs if *φ*(*ρ*_*y*_ > *θ*, *θ*) > 0. Finally. there is no synaptic change for *φ*(0, *θ*) = 0. Furthermore, by relating triplet-STDP to the BCM rule, the change in synaptic weight can be expressed as^39,40,56,57^

2 $$dG_c/dt = {\mathrm{ - }}A_{\mathrm{2}}^{\mathrm{ - }}\tau _{\mathrm{ - }}\rho _x\rho _y{\mathrm{ - }}A_{\mathrm{3}}^{\mathrm{ - }}\tau _{\mathrm{ - }}\tau _x\rho _x^2\rho _y + A_{\mathrm{2}}^ + \tau _ + \rho _x\rho _y + A_{\mathrm{3}}^ + \tau _ + \tau _y\rho _x\rho _y^2,$$

where ${A}^+_2$ and ${A}^-_2$ are the amplitude parameters of the paired term contribution for potentiation and depression, where a presynaptic spike triggered before and after a postsynaptic spike can induce LTP and LTD following the classical paired-STDP. The interval time between presynaptic spike and postsynaptic spike is not substantially longer than *τ*_+_ and *τ*_*−*_. As proposed in the literature^39,40,56^, the presence of a previous postsynaptic spike causes the potentiation contribution of triplet term *A*_3_^+^ in addition to the paired term in the ‘post-pre-post’ triplet. The interval between these two postsynaptic spikes should be in a time window of *τ*_*y*_. Similarly, for the depression contribution of the triplet term, $A^-_3$ and *τ*_*x*_ are the amplitude parameter and interval window of two postsynaptic spikes, respectively. All these parameters were extracted from our experiments on paired and triplet spikes (Supplementary Fig. 6 and Note 3). Following an approach reported in the literature^40^, the minimal triplet rule was considered by setting ${A}^+_2$  = 0 µS, ${A}^-_2$ = 0.02 µS, ${A}^+_3$ = 0.96 µS, *A*_3_^−^ = 0 µS, *τ*_+_ = 38 ms, *τ*_*−*_ = 30 ms, *τ*_*x*_ =16 ms, and *τ*_*y*_ = 0 ms as parameters in the present study (Supplementary Table 1). Additionally, the sliding threshold effect with different experienced *G*_0_ is a typical history-dependent property of the BCM learning rule, as mentioned previously. To simulate the sliding threshold effect, threshold θ differentiating potentiation and depression can be expressed as^39,40,54^

3 $$\theta\,{\mathrm{ = }}\,\left\langle {\rho _{\mathrm{y}}^p} \right\rangle \left( {A_{\mathrm{2}}^{\mathrm{ - }}\tau _{\mathrm{ - }}A_{\mathrm{2}}^{\mathrm{ + }}\tau _{\mathrm{ + }}} \right)/\left( {\rho _{\mathrm{0}}^pA_{\mathrm{3}}^ + \tau _ + \tau _y} \right),$$

where coefficient *ρ*_0_ and index *p* were set to 10 Hz and 2, respectively, for the calculations. Following the literature^39,40,56^, the dependence of ${A}^+_2$ and ${A}^-_2$ on the mean firing rate 〈*ρ*_*y*_〉 was introduced by setting ${A}^+_2$ → ${A}^+_2$ 〈*ρ*_*y*_^*p*^〉/*ρ*_*0*_^*p*^ and ${A}^-_2$ → ${A}^-_2$ 〈*ρ*_*y*_^*p*^〉/*ρ*_*0*_^*p*^. *ρ*_*y*_^*p*^ and *ρ*_*0*_^*p*^ denote the expectation of the *p*^th^ power of *ρ*_*y*_ and *ρ*_*0*_, respectively. For our experimental data, this dependence may be related to the experienced *G*_0_. As a result, the calculated curve of the BCM theory well fits the experimental data for each *G*_0,_ as illustrated in Fig. 4a, and the threshold sliding effect induced by tuning *G*_0_ is satisfactorily simulated. The calculated correlations of Δ*G* and the postsynaptic spike rate *ρ*_*y*_ allow the accurate simulation of neural networks for spatiotemporal patterns.

To extend the triplet-STDP-based BCM model to the application of spatiotemporal patterns, we adopted a two-layer neuromorphic feedforward network, as schematically shown in Fig. 4b. Rate-based orientation selectivity, as the typical input selectivity of spatiotemporal patterns, was numerically simulated using this network following BCM theory. The first layer of presynaptic neurons generate the presynaptic spikes in correspondence of the presentation of a visual pattern, whereas the second layer of postsynaptic neurons generate postsynaptic spikes upon excitation of the first layer. In our network, 81 presynaptic neurons (in a 9 × 9 array) and four postsynaptic neurons (with specific number *n* = 1, 2, 3, 4) were used as the input and output layers, respectively. As a result, there are 81 × 4 = 324 connections (i.e., synapses) between the two layers of the network. Generally, synapses are trained using presynaptic spikes from input neurons with spike rate *ρ*_*x*_ and a synchronous spike train from the postsynaptic neuron with rate *ρ*_*y*_. At each learning epoch, four patterns, each containing 81 pixels, with different orientation bars (i.e., 0°, 45°, 90°, and 135°) and four random noise patterns were presented to the presynaptic neurons. The presentation of the orientation patterns and noise patterns had equal probability. For the orientation patterns, only the 9 pixels of these four orientation bars had a high spike rate with a Poisson distribution (i.e., 〈*ρ*_*x*_〉 = 40 Hz_,_ black), whereas the other 72 pixels had a low spike rate with a Poisson distribution (i.e., 〈*ρ*_*x*_ 〉 = 10 Hz_,_ white). For the noise inputs, nine pixels were randomly selected with a high spike rate, and the others had a low spike rate. Four ${\rho}^n_y$ corresponded to these four postsynaptic neurons. Every ${\rho}^n_y$ was updated in real time after each pattern or noise according to synaptic weight *G*_*m*_^*n*^ in the last pattern or noise using the equation $\rho _y^n = \mathop {\sum}\nolimits_{m = 1}^{81} {\rho _{x,m}} \times G_m^n$ (*m* = 1, 2, … 81, *n* = 1, 2, 3, 4)^58,59^. To achieve the selectivity of multiple orientations, the four *ρ*_*y*_^*n*^ were compared after each pattern or noise submission. Only the specific postsynaptic neuron with the maximal ρ_y_^n^ could send the fire postsynaptic spikes following the winner-take-all rule, thereby modifying the synaptic weight combined with presynaptic spikes (Supplementary Fig. 7). Correspondingly, the threshold θ^n^ was also updated with *ρ*_*y*_^*n*^ according to Eq. (3).

All the synapses started from a stochastic initial state with low *G*_0_. Considering the 1st postsynaptic neuron as example, the *G*^1^ evolution of the extracted synapses only for the four orientations with the learning epoch is shown in Fig. 4c. During the increasing epochs, once a specific orientation was randomly selected by the 1st postsynaptic neuron with strong potentiation, there would be a corresponding higher *ρ*_*y*_^1^ > *θ*^1^. Meanwhile, the relatively low *ρ*_*y*_^1^ < *θ*^1^ was introduced for the other three orientations, thereby leading to depression. Figure 4d shows the ${\rho}^1_y$ evolution of the four orientation bars as a function of the learning epochs. From the results of Fig. 4c, d, *G*^1^ and *ρ*_*y*_^1^ of the 45° orientation bar clearly increase after thousands of learning epochs, which suppressed the values of the other three orientations. Eventually, the orientation of 45° was selected for the 1st postsynaptic neuron, as shown in Fig. 4e–I, in which the conductance of the nine pixels for the 45° orientation bar was obviously higher than the others. Simultaneously, although the other three orientations were suppressed in the first postsynaptic neuron, they were selected without supervision by the other three postsynaptic neurons, thereby generating strong potentiation with higher *ρ*_*y*_^*n*^ (*n* = 2, 3, 4). As shown in Fig. 4e-II–e-IV, the orientations of 0°, 90°, and 135° were selected by the 2nd, 3rd, and 4th postsynaptic neurons, respectively, which verifies the feasibility of complete rate-based orientation selectivity in this study. The above results suggest the potential application of the spatiotemporal patterns in our memristors.

## Discussion

We demonstrated a generalized triplet-STDP-based BCM learning rule using a WO_3−x_-based second-order memristor. Compared with the BCM rules realized by common rate-based presynaptic spikes, the EDE region missing in previous studies was found in our experimental data. A typical threshold sliding effect that depended on the learning history was also obtained. Furthermore, rate-based orientation selectivity was demonstrated in a feedforward network based on the generalized BCM framework in our memristors by simulation, which indicated its potential feasibility for high-order spatiotemporal patterns. It is noted that there are still certain limitations to a full implementation of the BCM learning at the synaptic level using memristors. For instance, the device physics and signal design may bring differences from the biological synapse, such as the spike-timing region, LTP/LTD window, and specific biological features. Further studies are still required to solve these above limitations toward a fully bio-mimetic BCM rule. It is believed that our study makes a progress towards the biorealistic mimicking of BCM learning rules in memristive synapses and paves the way for the application of memristors to spatiotemporal patterns in the future.

## Methods

### Device fabrication

Memristors with a Pt/WO_3−x_/W sandwich structure were fabricated on SiO_2_/Si substrates and patterned into a crossbar array with a junction area of 50 × 50 µm^2^ using a metal mask, as shown in Fig. 1b. Both the W bottom electrode and WO_3−x_ film with thicknesses of 100 and 80 nm were subsequently prepared by RF sputtering using a metal W target. The WO_3−x_ film was grown under a gas pressure of 2 Pa (Ar:O_2_ = 3:1) at 200 °C. The 80-nm-thick Pt top electrode was fabricated through electron-beam evaporation.

### Electrical measurements

Memristive properties were measured using a self-built test system comprising a sourcemeter (2636A, Keithley), arbitrary function generator (3390, Keithley), oscilloscope (TDS 2012B, Tektronix), and probe station (TTPX, Lake Shore). The positive direction of the bias voltage was defined such that the current flowed from the top to the bottom electrode. To measure the EPSC, the memristor was connected with a load resistor R_load_ of 1 MΩ in series, and the voltage drop across the R_load_ was monitored by an oscilloscope. Then, the monitored voltage was converted to the current flowing through the memristor. To implement pair- or triplet-STDP, each pre- or postsynaptic spike applied to the top or bottom electrode was composed of a pair of pulses with amplitude *V*^+^/*V*^−^ = 2 V/–2 V and a width of 50 ms. The initial and final conductance states of the device (*G*_*i*_ and *G*_final_) were readout using a small pulse [0.2 V, 50 ms] before and after applying the programmable pulses, and the conductance change was defined as Δ*G*_*c*_ = *G*_final_ − *G*_i_. For the experienced *G*_0_, Δ*G*_*c*_ = *G*_final_ − *G*_0_. Both the writing and reading of the memristor were performed in pulse mode.

## Supplementary information

Supplementary Information

## Data Availability

The data that support the plots within this paper and other findings of this study are available from the corresponding author upon reasonable request.

## Source Data

Source Data
