# Supplementary material for: Toward a generalized Bienenstock-Cooper-Munro rule for spatiotemporal learning via triplet-STDP in memristive devices
Source: Nat Commun. 2020 Mar 20;11:1510. doi: 10.1038/s41467-020-15158-3 (PMC7083931; doi:10.1038/s41467-020-15158-3)
Supplement: Supplementary file 1 — Supplementary Information [file 41467_2020_15158_MOESM1_ESM.pdf]

Supplementary Information

Toward a generalized Bienenstock-Cooper-Munro rule for spatiotemporal learning via triplet-STDP in memristive devices

Wang et al.

Supplementary Figures and Supplementary Notes

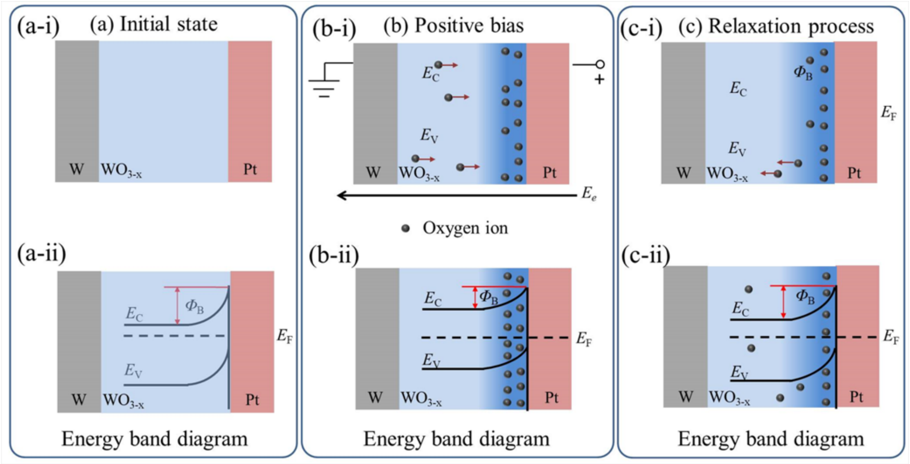

Supplementary Figure 1| Operating mechanism of the WO_{3-x} second-order memristor. Schematic diagram of the memristive mechanism shows the energy band alignment of the Pt/WO_{3-x} Schottky interface at different memristive states, including (a-i) the initial state, (a-ii) energy band diagram of the initial state, (b-i) oxygen ions migrating under positive bias, (b-ii) energy band diagram after the voltage is removed, (c-i) relaxation process: back diffusion of oxygen ions, and (c-ii) energy band diagram after the voltage is removed for a long time.

Supplementary Note 1. The modulation of the Schottky barrier height Φ_B at the Pt/WO_{3-x} interface is responsible for the memristive behavior in the present device.[1,2] The Schottky barrier height can be expressed as

$$\Phi_B = E_F - E_{Fm}, \tag{1}$$

where E_F and E_{Fm} are the Fermi level of WO_{3-x} and Pt, respectively. The drift of oxygen ions toward the Pt electrode occurs under positive bias, thereby leading to

their accumulation at the Pt/WO_{3-x} interface. As a result, the Fermi level of WO_{3-x} films falls following [3]

$$E_F = (\hbar^2 / 2m^*)(3\pi^2 n_0)^{2/3}, \tag{2}$$

where n_0 is the electron concentration of WO_{3-x} film on the surface, which decreases because of the accumulation of oxygen ions. When the positive voltage is removed, the reduction of Schottky barrier height Φ_B of Pt/WO_{3-x} still exists as illustrated in Supplementary Figure 1 (b-ii), thereby increasing the device conductance**.** After the bias voltage is removed, there is back-diffusion of oxygen ions because of the concentration gradient with the help of the built-in electric field, which results in a partial recovery of barrier Φ_B and thus conductance relaxation, as illustrated in Supplementary Figure 1 (c). When a negative bias is applied to the Pt electrode, the Schottky barrier is enhanced and the device conductance is reduced. In the first-order memristor, the conductance change (ΔG_c) is solely determined by the applied voltage on the device as follows [4,5]:

$$dG_c / dt = f(G_c, V, t), \tag{3}$$

where G_c is the device conductance (weight) state variable and V is the applied voltage. By contrast, the second-order memristor includes one more state variable in the mathematical equation [4,5], that is,

$$dG_c / dt = f(G_c, G_m, V, t), \tag{4}$$

where G_m is the second-order variable that can affect the modulation of G_c. In the present work, G_c can be ascribed to the Schottky barrier as discussed previously,

whereas G_m represents oxygen ion mobility in the film.[6] Therefore, the spontaneous decay of conductance G_c observed in Figure 1(e) manifests the diffusion of oxygen ions, which is a clear indicator of the second-order memristor.

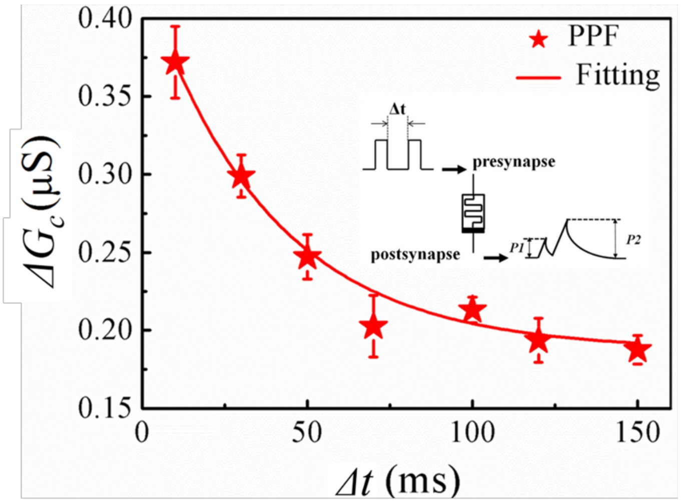

Supplementary Figure 2| Emulation of the PPF function. The PPF magnitude depends on the interval time of paired pre-spikes. As reported in the literature,[1,6,7] the peaks of both EPSCs were measured (*P1* and *P2*) as illustrated in the inset. The PPF was calculated by $\Delta G_c = (P2\text{-}P1)/P1 \times 100\%$. The potentiation effect exponentially weakens with increasing interval time. The decay process can be fitted by a double exponential function.

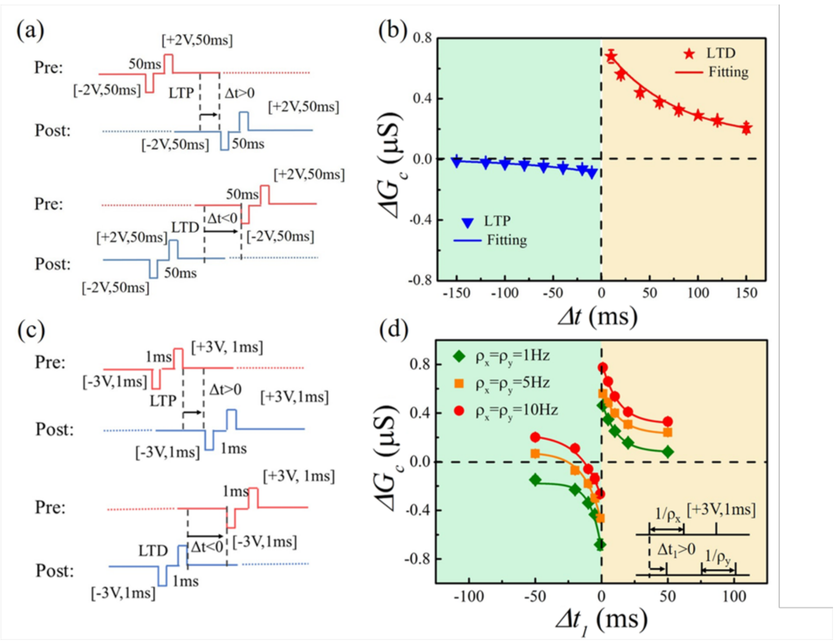

Supplementary Figure 3| Demonstration of paired-STDP in the WO_{3-x} memristor. (a) Specific signal design of pre- and postsynaptic spikes. The symmetrical signals include pulses with amplitude V^{+}/V^{-} = 2V/−2 V and a duration of 50 ms, and a sufficient interval of 50 ms between V^{+} and V^{-} is introduced to ignore the effect of V^{+} on V^{-} according to the decay time of the EPSC, which was discussed in previous work.[1-2,8] This signal design was adopted to realize triplet-STDP. (b) STDP rule of long-term plasticity demonstrated for paired spikes and G_0 = 0.1 μS. (c) The interval time of 50ms can be reduced to 1 ms using a pulse with higher amplitude and shorter duration. A pulse [3V, 1ms] was used for stimulations as an example. It is also suitable to realize triplet-STDP. (d) Triplet-STDP was demonstrated using the signal design of (c). Comparing to the triplet results in Figure 3 (f), a different time lag and ΔG_c were obtained because of the different pulse stimulation.

Supplementary Note 2. The demonstration of paired-pulse facilitation (PPF) and spike-timing-dependent plasticity (STDP) in our memristor are related to the effect of

spike timing on the oxygen ions diffusion (i.e., G_m). The drift and accumulation of oxygen ions at the Pt/WO_{3-x} interface can induce the conductance increase, whereas the subsequent diffusion of oxygen ions reduces its accumulation, which leads to conductance decay, as illustrated in Figure 2 (a). Because the intensity of EPSC would gradually decay to zero within hundreds of ms after the stimulation. If another stimulation comes before the first EPSC disappears completely, their overlap can effectively suppress the diffusion of oxygen ions. This promotes the more effective accumulation of oxygen ions on the Pt/WO_{3-x} interface, which leads to a larger conductance change. For a more detailed explanation, refer to our previous work.[8] Therefore, shorter intervals (higher frequency) induced a larger conductance increase, as demonstrated by the PPF function (Supplementary Figure 2). Additionally, the conversion from short-term plasticity to long-term plasticity can be realized, as experimentally demonstrated in Figures 2 (b) and (c). Therefore, the interval-dependent feature is still applicable for implementing the long-term STDP and triplet-STDP by designing the operation signal as shown in Supplementary Figure 3 (a).

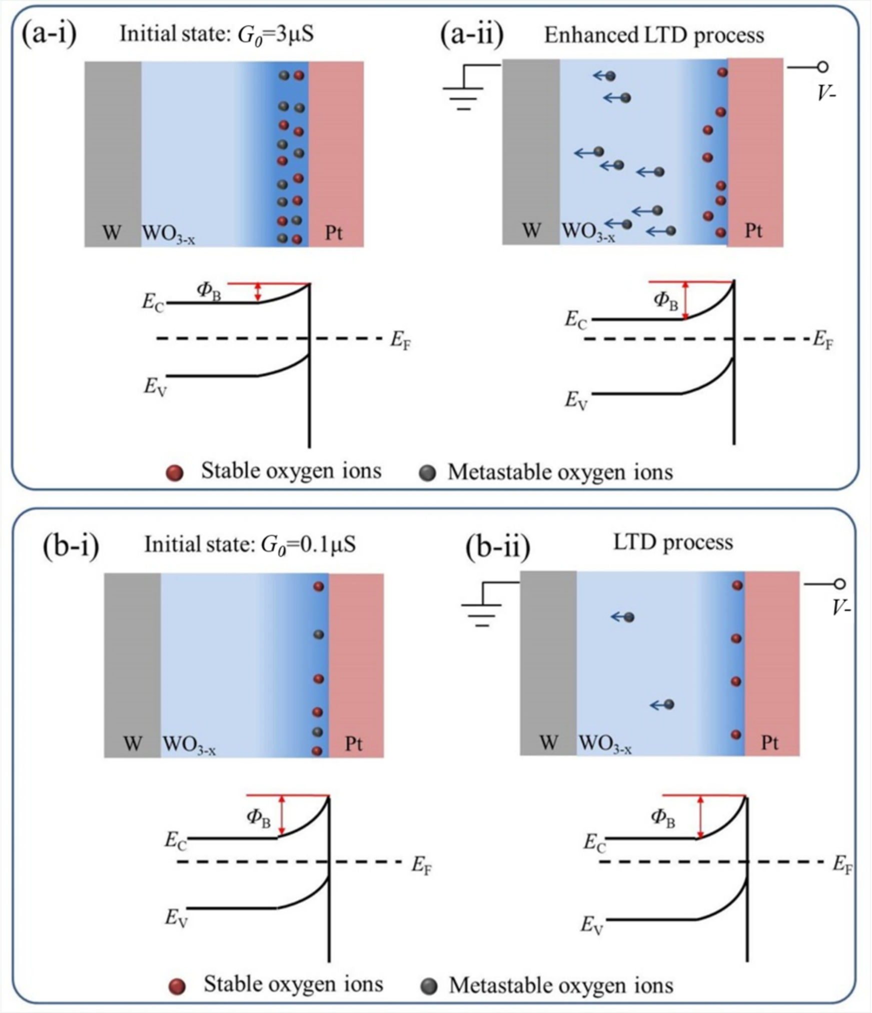

Supplementary Figure 4| Explanation for the EDE of the LTD process with high G_0. (a-i) Initial state and (a-ii) enhanced LTD process in the case of a relatively high experienced G_0 = 3.0 μS. (b-i) Initial state and (b-ii) LTD process for a low experienced G_0 = 0.1 μS. The forgetting effect of the experienced G_0 is generally related to metastable oxygen ions (e.g., unstable interstitial oxygen ions) with a lower energy barrier after stimulation. [8] During the LTD process, the back migration of oxygen ions increases the Fermi level of WO_{3-x}, which increases barrier height Φ_B. As illustrated in Supplementary Figure 4 (a-i) and (b-i), more metastable oxygen ions exist at the Pt/WO_{3-x} interface for higher experienced G_0= 3μS than for G_0= 0.1μS. Correspondingly, under the stimulation of the LTD process, the depression effect be

enhanced (i.e., more oxygen ions can be driven back) than for $G_0 = 0.1\mu S$, as shown in Supplementary Figures 4 (a-ii) and (b-ii). This enhanced LTD process can result in a larger modification of the Schottky barrier (i.e., larger $\Delta\Phi_B$) and conductance change ΔG_c for depression; that is, the appearance of an EDE region.

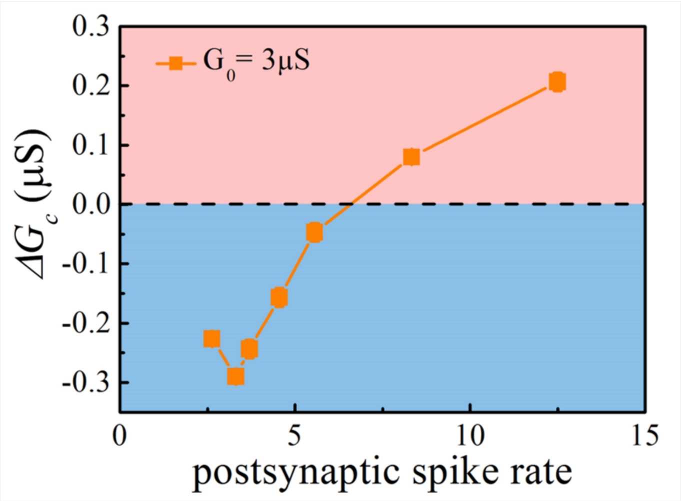

Supplementary Figure 5| Triplet-STDP-based BCM learning rule obtained using asymmetrical spike timing. By extracting data from quadrant II of Figure 3(d) with a fixed spike-timing difference of $|\Delta t_1| - |\Delta t_2| = 20$ ms, similar BCM learning rules with the EDE are demonstrated.

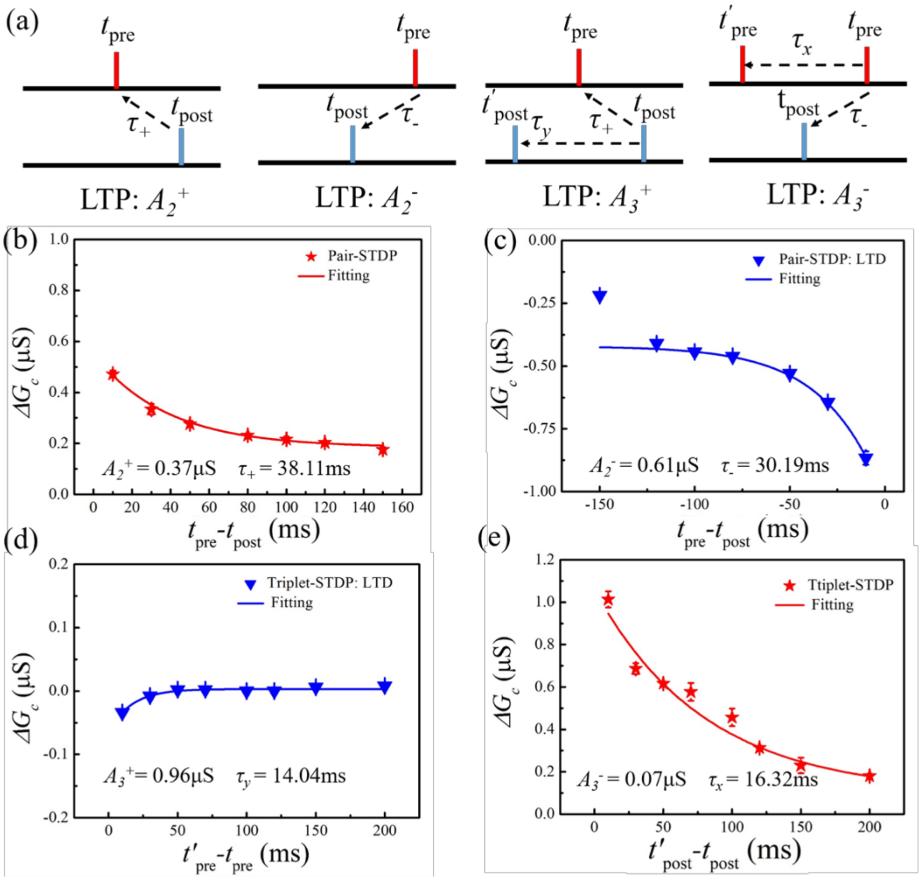

Supplementary Figure 6| Parameter fitting of triplet learning rules. (a) Schematic description of the paired term and triplet term that contribute to LTP/LTD. (b-e) Parameters fitting in paired-STDP and triplet-STDP. For the paired term, its contribution follows the classical paired STDP rule,[9-13]

$$\Delta G_c = \begin{cases} \Delta G_c^{\ +} = A_2^{\ +} \exp(-\Delta t/\tau_+) & \text{if } \Delta t > 0, \\ \Delta G_c^{\ -} = -A_2^{\ -} \exp(\Delta t/\tau_-) & \text{if } \Delta t < 0. \end{cases} \quad (5)$$

A presynaptic spike triggered before a postsynaptic spike can induce LTP with an amplitude parameter of A_2^+, if the interval time $\Delta t = t_{post} - t_{pre}$ is not substantially longer than τ_+,where, t_{pre} and t_{post} denote the moment of the presynaptic spike and postsynaptic spike. Accordingly, A_2^- and τ_- are the amplitude parameter and time

constant for LTD, respectively, as shown in Supplementary Figure 6 (a-ii). As proposed in the literature,[9-10] the presence of a previous postsynaptic spike causes the potentiation contribution of triplet term A_3^+ in addition to the paired term in the 'post-pre-post' triplet, as shown in Supplementary Figure 6 (a-iii). The interval of these two postsynaptic spikes should be in a time window of τ_y. For triplet term induced depression, a previous presynaptic spike makes the contribution with the amplitude parameter of A_3^- in the 'pre-post-pre' triplet, as shown in Supplementary Figure 6 (a-iv). The interval of these two postsynaptic spikes should be in a time window of τ_x. The mathematical representation of the triplet learning rule is given by[9-12]

$$\Delta G_c = \begin{cases} \Delta G_c^+ = \exp(-\Delta t/\tau_+)(A_2^+ + A_3^+(-\Delta t_o/\tau_y)) \\ \\ \Delta G_c^- = -\exp(\Delta t/\tau_-)(A_2^- + A_3^-(\Delta t_r/\tau_x)) \end{cases} \quad (6)$$

where $\Delta G_c = \Delta G_c^+$ if $t = t_{post}$ represents the case of the 'post-pre-post' triplet; and $\Delta G_c = \Delta G_c^-$ if $t = t_{pre}$, represents the case of the 'pre-post-pre' triplet. $\Delta t = t_{post} - t_{pre}$ is the interval time between the presynaptic spike and postsynaptic spike. t'_{pre} and t'_{post} are the moments of the previous presynaptic spike and postsynaptic spike in the 'pre-post-pre' triplet or 'post-pre-post' triplet, respectively. $\Delta t_o = t'_{post} - t_{post}$ and $\Delta t_r = t'_{pre} - t_{pre}$ are the interval times between two postsynaptic spikes and presynaptic spikes. Parameters A_2^+, A_3^+, A_2^-, and A_3^- are the potentiation and depression amplitude parameters, whereas τ_+, τ_-, τ_x, and τ_y are the time constants as addressed previously.

Supplementary Note 3. To ascertain the parameters, ΔG_c as a function of $\Delta t = t_{post} - t_{pre}$, $\Delta t_o = t'_{post} - t_{post}$, and $\Delta t_r = t'_{pre} - t_{pre}$ was experimentally measured in Supplementary

Figure 6 (b-e), and represents the paired-STDP and triplet-STDP learning rules, respectively. The experienced G_0 = 3.0 μS was considered in the measurements. From the fitting of experimental data in Supplementary Figures 6 (b) and (c), the parameters of the paired term, including A_2^+, A_2^-, τ_+, and τ_-, can be confirmed. Furthermore, these paired term parameters were taken into account in equation (6) for fitting the experimental data in Supplementary Figures 6 (d) and (e). Eventually, the parameters of the triplet term, including A_3^+, A_3^-, τ_x, and τ_y, were extracted. All these parameters are summarized in Supplementary Table 1. Furthermore, as proposed by Pfister et al.,[9] only some of the parameters are really necessary in the model for fitting visual cortex data. Therefore, a minimal model was widely used for the triplet learning rule by making $A_2^+ = 0$ and $A_3^- = 0$. For the details, refer to Pfister et al.'s work.[10] Similar considerations were also reported in other studies.[10-11] In the present work, we also adopted the minimal model for simulations, whose parameters are also shown in Supplementary Table 1.

Supplementary Table 1. Parameters extracted from Figure S6, optimized using the minimal triplet model.

Parameters	A_2^+ (μS)	A_2^- (μS)	τ_+ (ms)	τ_- (ms)	A_3^+ (μS)	A_3^- (μS)	τ_x (ms)	τ_y (ms)
Experimental data	0.37	0.61	38.11	30.19	0.96	0.07	16.32	14.04
Minimal triplet model	0	0.02	38	30	0.96	0	16	0

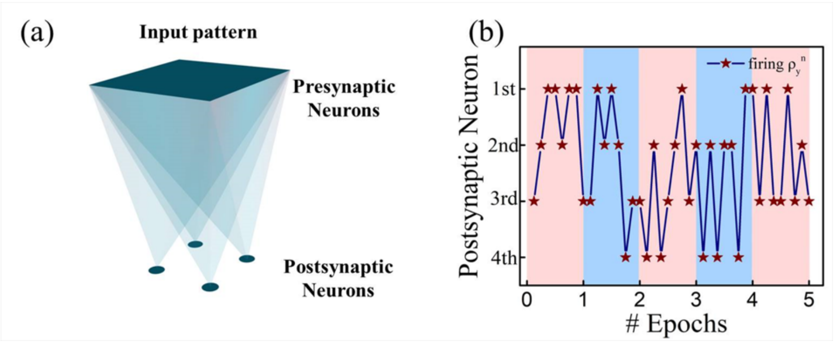

Supplementary Figure 7| $\rho_y{}^n$ fires following the winner-take-all rule for each pattern or noise. (a) The network includes 81 presynaptic neurons and four postsynaptic neurons. (b) Each epoch contains four orientation patterns and four noises. After each pattern or noise, $\rho_y{}^n$ was calculated according to the synaptic weight $G_m{}^n$ in the last pattern or noise using $\rho_y{}^n = \sum_{m=1}^{81} \rho_{x,m} \times G_m^n$ (m = 1, 2, …81, n =1, 2, 3, 4). Then, only the specific postsynaptic neuron with the maximal $\rho_y{}^n$ could fire postsynaptic spikes. Supplementary Figure 7 (b) shows the evolution of $\rho_y{}^n$ firing in the initial five epochs.

Supplementary References

1. Lin, Y. et al. Transferable and flexible artificial memristive synapse based on WO_x schottky junction on arbitrary substrates. *Adv. Electron. Mater.*, 1800373 (2018).
2. Lin, Y. et al. Analog-digital hybrid memristive devices for image pattern recognition with tunable learning accuracy and speed. *Small Methods*,1900160(2019).

3. Look, DC. et al. Self-compensation in semiconductors: The Zn vacancy in Ga-doped ZnO. *Physical Review B* 2011, 84(11): 115202.

4. Kim, S. et al. Experimental demonstration of a second-order memristor and its ability to biorealistically implement synaptic plasticity. *Nano letters* **15**, 2203-2211 (2015).

5. Pershin, YV. & Di, VM. Neuromorphic, digital, and quantum computation with memory circuit elements. *P. IEEE* 100, 2071-2080 (2012).

6. Du, C., Ma, W., Chang, T., Sheridan, P. & Lu, WD. Biorealistic implementation of synaptic functions with oxide memristors through internal ionic dynamics. *Adv. Funct. Mater.* 25, 4290-4299 (2015).

7. Zucker, RS. & Regehr, WG, short-term synaptic plasticity, *Annu. Rev. Physiol.*, 64, 355 (2002).

8. Wang, ZQ. et al. Synaptic learning and memory functions achieved using oxygen ion migration/diffusion in an amorphous InGaZnO memristor. *Adv. Funct. Mater.* **22,** 2759-2765 (2012).

9. Pfister, J-P. & Gerstner, W. Triplets of spikes in a model of spike timing-dependent plasticity. *J. Neurosci.* **26**, 9673-9682 (2006).

10. Gjorgjieva, J., Clopath, C., Audet, J. & Pfister, J-P. A triplet spike-timing–dependent plasticity model generalizes the Bienenstock-Cooper-Munro rule to higher-order spatiotemporal correlations. *Pro. Natl Acad. Sci.USA.* **108**, 19383-19388 (2011).

11. Azghadi, MR., Al-Sarawi, S., Iannella, N. & Abbott, D. Design and implementation of BCM rule based on spike-timing dependent plasticity. *In Neural Networks (IJCNN)*, International Joint Conference on 1-7 (IEEE 2012).
12. Cai, W., Ellinger, F. & Tetzlaff, R. Neuronal synapse as a memristor: Modeling pair-and triplet-based STDP rule. *IEEE transactions on biomedical circuits and* systems 9, 87-95 (2014).
13. Ascoli, A., Corinto, F. & Tetzlaff, R. Generalized boundary condition memristor model. *International Journal of Circuit Theory and Applications* 44, 60-84 (2016).
